# A simplified network topology for fruit detection, counting and mobile-phone deployment

**DOI:** 10.1371/journal.pone.0292600

**Published:** 2023-10-09

**Authors:** Olarewaju Mubashiru Lawal, Shengyan Zhu, Kui Cheng, Chuanli Liu

**Affiliations:** Sanjiang Institute of Artificial Intelligence & Robotics, Yibin University, Yibin, Sichuan, China; Hainan Normal University, CHINA

## Abstract

The complex network topology, deployment unfriendliness, computation cost, and large parameters, including the natural changeable environment are challenges faced by fruit detection. Thus, a Simplified network topology for fruit detection, tracking and counting was designed to solve these problems. The network used common networks of Conv, Maxpool, feature concatenation and SPPF as new backbone and a modified decoupled head of YOLOv8 as head network. At the same time, it was validated on a dataset of images encompassing strawberry, jujube, and cherry fruits. Having compared to YOLO-mainstream variants, the params of Simplified network is 32.6%, 127%, and 50.0% lower than YOLOv5n, YOLOv7-tiny, and YOLOv8n, respectively. The results of mAP@50% tested using test-set show that the 82.4% of Simplified network is 0.4%, -0.2%, and 0.2% respectively more accurate than 82.0% of YOLOv5n, 82.6% of YOLOv7-tiny, and 82.2% of YOLOv8n. Furthermore, the Simplified network is 12.8%, 17.8%, and 11.8% respectively faster than YOLOv5n, YOLOv7-tiny, and YOLOv8n, including outperforming in tracking, counting, and mobile-phone deployment process. Hence, the Simplified network is robust, fast, accurate, easy-to-understand, fewer in parameters and deployable friendly.

## Introduction

### Motivation

Having introduced artificial intelligence (AI) to promote fruit production through agricultural sector, labor costs have become inexpensive, labor-intensive processes have been cut, timeliness has increased, and fruit quality and quantity have increased (Sa et al. [1]). Fruit detection with computer vision using deep learning is a technique used to localize and classify targets in an image or video. It has widely been applied and researched for monitoring, picking, harvesting, yield prediction, estimation, counting, production, and so on according to Koirala et al. [2], Lawal [3–5], Qiao et al. [6]. Regardless, the complex network topology, deployment unfriendliness, and large parameters, including the natural changeable environment such as occlusion, illumination, similar background appearance, nonstructural fields (Lawal et al. [7]) among others are some of the limitations encountered by fruit detection. Therefore, this paper designed a simplified network topology with fewer parameters and improved performance based on YOLOv8 framework in order to real-time detect, track, and count fruit targets in the natural environment. The contributions of this paper are to:

1. create a robust fruit image dataset of dense targets with complex natural environment.
2. introduce only convolution, maxpooling, and feature concatenation into backbone network, and replace the C2f of YOLOv8 head network with convolution to develop a simplified network topology for fruit detection, tracking and counting.
3. produce a Simplified network topology that is robust, fast, accurate, easy to understand, fewer in parameters and deployable.
4. compare performance of the Simplified network topology to YOLOv5n, YOLOv7-tiny, and YOLOv8n, including other YOLO variants.

### Related work

The advent of YOLO framework and continuous growth has revolutionized fruit detection with computer vision. YOLO became famous for its single-stage object detector approach, which was especially used to improve detection speed. From YOLOv3 launched by Redmon and Farhadi [8], Gai et al. [9] improved the YOLOv3-tiny method, and reported average precision (AP) of 95.56% and speed of 35.5 frames per second (fps) for real-time object detection. The FL-YOLOv3-tiny developed by Tan et al. [10] noted 10.9% AP and 29% fps increase from the YOLOv3-tiny for underwater object detection, and Cong et al. [11] with the developed MYOLO achieved a mean AP of 97.03% and a speed of 50.6 fps for mushrooms detection. Fu et al. [12] reached AP of 90.05% and a speed of 29.4 fps for kiwifruits detection in orchard, while Zheng et al. [13] reported AP of 88.8% and speed of 40 fps on muskmelon fruit detection. Lawal [3] and Liu et al. [14] revealed that an improved YOLOv3 can solve the factors of fruit detection. As a build on YOLOv3 toward detection performance improvement, YOLOv4 was released by Bochkovskiy et al. [15]. Latha et al. [16] published mean AP of 51% and speed of 55.6 fps for fruits and vegetables detection using YOLOv4-tiny. Meanwhile, the YOLOv4-tiny was modified by Parico et al. [17], which reported a speed of more than 50 fps and AP of 94.19% for real-time pear fruit detection and counting, and Tang et al. [18] achieved 92.07% of AP and speed of 32.3 fps to detect each camellia oleifera fruit targets in orchard. The GCS-YOLOv4-tiny proposed by Mei-Ling and Yang [19] revealed AP of 93.42% with 17.45% increase from YOLOv4-tiny to detect different growth stages of fruits. The quest for a faster detection speed led to the birth of YOLOv5 by Jocher et al. [20]. The YOLO-Jujube introduced by Xu et al. [21] to detect jujube fruit automatically for ripeness inspection achieved AP of 88.8% and a speed of 245 fps, and Yan et al. [22] recorded AP of 86.75% and a speed of 66.7 fps to detect apple fruit targets based on improved YOLOv5. To detect a dragon fruit in the natural environment, Zhang et al. [23] realized AP of 97.4% with the incorporation of ghost network (Han et al. [24]) into YOLOv5. Qiao et al. [6] introduced ShuffleNetv2 network proposed by Ma et al. [25] into YOLOv5 for a counting method of red jujube, which noted AP of 94% and speed of 35.5 fps. Gai et al. [26] documented a cherry detection of 0.08 and 0.03 for F_1_-score higher than the YOLOv4 and YOLOv5, respectively, with the proposed YOLOv5s-cherry. Lawal et al. [27] used feature concatenation with coordinate attention mechanism (CAM) (Hou et al. [28]) to improved YOLOv5s for fruit detection. The improved YOLOv5s based on mean AP and speed recorded 0.6% and 10.4% respectively higher than the original YOLOv5s network. In order to achieve a lightweight algorithm for strawberry fruit detection, Lawal [29] developed YOLOStrawberry by combined lightweight networks. The obtained 3.37 MB of weight-size, 89.7% of AP and 137 fps of speed time outperformed YOLOv5n, including some other lightweight YOLO-variants. The recent YOLOv7 designed by Wang et al. [30] was declared to have surpassed YOLOv4 and YOLOv5 in detection performance. For this reason, Zhang et al. [23] applied both YOLOv7 and YOLOv7-tiny for dragon fruit detection, and respectively realized AP of 95.6% and 96.0%. Additionally, Chen et al. [31] improved the YOLOv7 using a CBAM (Convolutional Block Attention Module) for citrus detection, achieving AP of 97.29% and a speed of 14.4 fps. Nevertheless, the aforementioned YOLO variants’ networks are anchor-based detectors, leading to the latest introduction of YOLOv8 by Jocher et al. [32], which is anchor-free. The anchor-free detectors directly predict the offsets of a point to its outside boundaries, unlike anchor-based that predict the offsets based on predefined anchor. YOLOv8 was designed to be flexible, easy to use, fast and accurate with new features compared to previous YOLO versions. However, it is still in the development stage and yet to be experimented for fruit detection.

Furthermore, the YOLO-mainstream variants, including other existing YOLO variants are associated with complex network topology. These networks are very difficult to understand, particularly when the operations within each downsampling block of backbone and head network possesses more convolution, addition, concatenation, maxpooling, split, or attention mechanisms, leading to a larger parameter. Due to this, it becomes a major challenge to achieve a faster detection speed and friendly deployment for low-power computing devices, i.e., it is hard to convert the network from one format, such as PyTorch to torchscript, ncnn, tensorrt, onnx, openvino, or tflite. Thus, it is necessary to investigate and address this limitation using a simplified network topology that is easy to understand, deployment friendly, fewer in parameters, accurate, fast, and can handle complex conditions.

The remaining part of this paper is organized as the second section provide methodology that involves image dataset, Simplified network, experiment and evaluation metrics. The third section describes the results and discussion, and the fourth section summarizes the conclusions and future plans.

## Methodology

### Dataset construction

**Fig 1:**
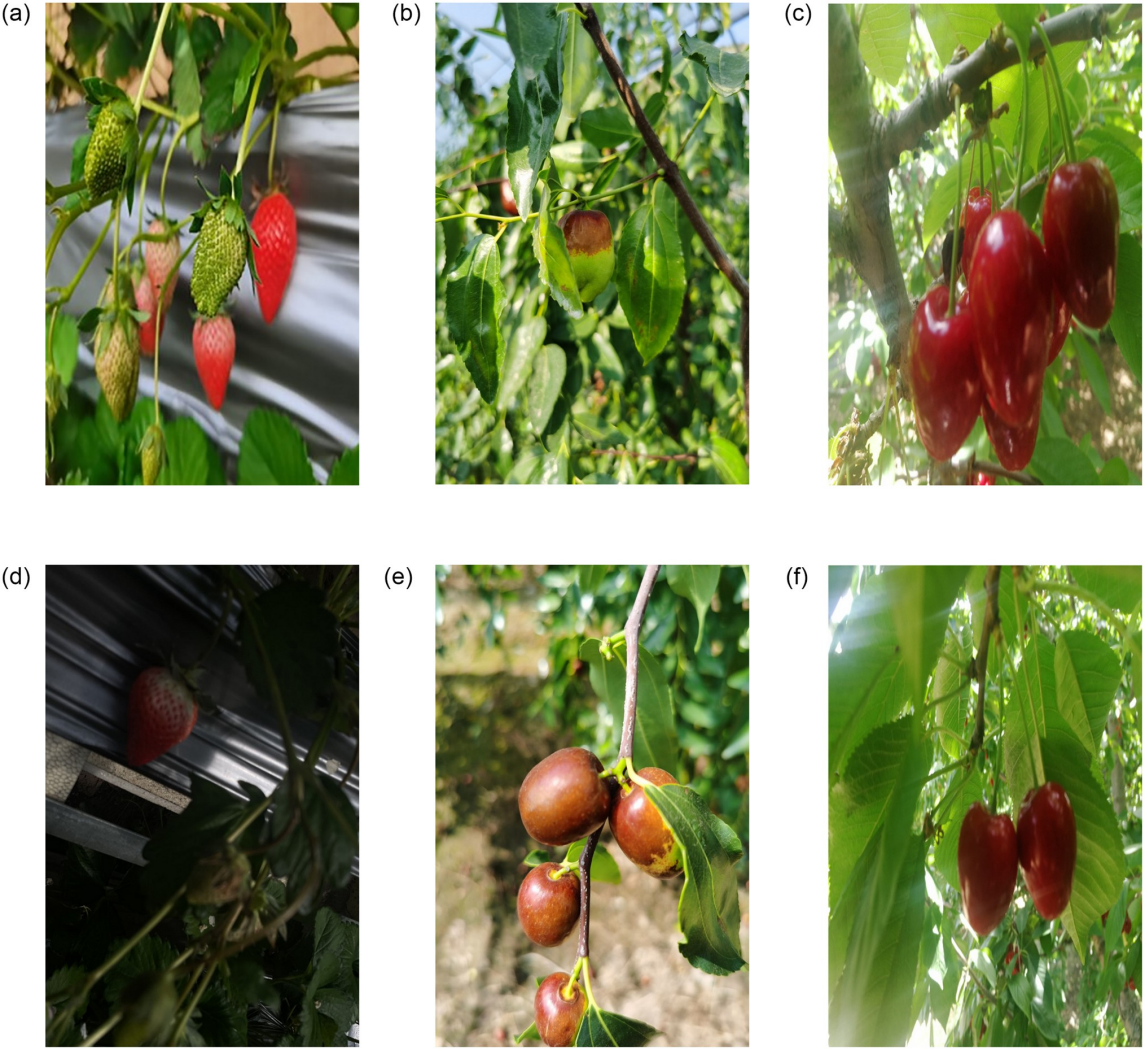
Obtained images under different natural environment (a) branch occlusion, (b) leaf occlusion, (c) overlap, (d) back light, (e) front light with soil background, and (f) side light.

The images of strawberry, jujube and cherry fruit used for this research work were captured from different locations within greenhouse and orchard in Jinzhong, Shanxi, China using a digital camera, Huawei mate30pro and mate40pro of 3968×2976, 1904×4096 and 2736×3648 pixels resolution respectively in morning, noon and afternoon with constantly changing distance. This field site requires no permits to obtain images of fruits because it is open to the public. The captured images under complex natural environment contain images with dense targets, branch occlusion, fruit clusters, leaf occlusion, overlap, back light, branches occlusion, earth background, similar background, sky background, front light, side light and other natural scenes, and saved in JPG format. Some of the obtained images are displayed in Fig 1.

Table 1 provides the dataset construction details, where valid provides an evaluation of a model fit on the train, and test as an unseen dataset is used to evaluate the level of model robustness. A total of 1350 images of strawberry, 1959 images of jujube and 948 images of cherry fruit were obtained, and randomly divided into 80% of train, 15% of valid, and 5% of test to create 3315, 681 and 261 images, respectively for network training and testing. Having labeled the ground truth bounding boxes of each image manually using labelImg tool while disregarding the complex nature of the image, a total of 39760, 6004 and 2579 boxes were created from images of train, valid, and test, respectively. The obtained labeled files were saved in the YOLO text format, which takes target class, coordinates, height and width. Again, recorded video of strawberry, jujube and cherry fruits saved in mp4 format were taken to investigate real-time detection speed, tracking and counting of detected fruit targets.

**Table 1:** Dataset construction details.

Divide	Strawberry	Jujube	Cherry	Images	Boxes
train	1082	1569	664	3315	39760
valid	199	292	190	681	6004
test	69	98	94	261	2579
**Total**	1350	1959	948	4257	48343

### Simplified network topology

**Fig 2:**
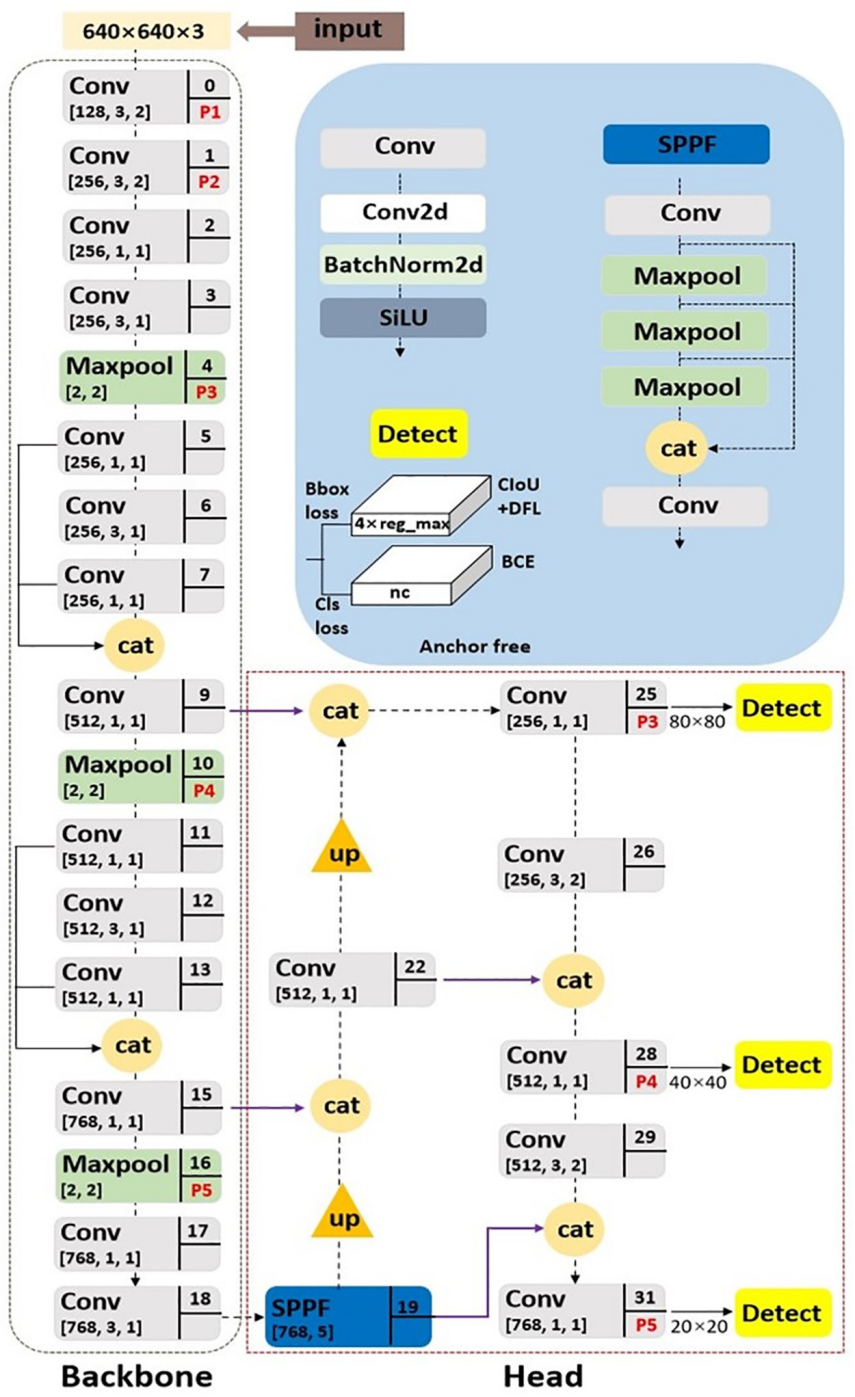
Simplified network topology for fruit detection.

The simplified network proposed for fruit detection is shown in Fig 2. It is builds upon YOLOv8n method, which contains the input, backbone network, and head network (neck and detect). Being an anchor-free network, the input integrates mosaic data augmentation and adaptive images scaling of 0.33 depth and 0.50 width. The backbone is simply a convolutional neural network adopted to accumulate fine-grained images, and for feature maps extraction as displayed in Fig 2. It comprises of Conv, Maxpool, feature concatenation and SPPF. Conv is a convolution activated with SiLU (Stefan et al. [33]) function immediately after batch normalization. Maxpool is used for downsampling of feature map, and feature concatenation enables information sharing between complementary features of low and high-layer. Eq (1) defined the feature concatenation O, where A is low-layer, B is high-layer, C is channels, H is height and W is width. SPPF increases accuracy and enable a faster detection according to Jocher et al. [20]. It enables feature enhancement by concatenation between the Conv and three-Maxpool as depicted in Fig 2. The decoupled head, which contains the neck and detect network outputs the class probability, score, and position of the bounding box surrounding the fruit target using anchor-free split. The neck combines path aggregation network (PAN) by Liu et al. [34] and feature pyramid network (FPN) by Lin et al. [35] for multiscale feature fusion collected from backbone. Neck includes upsampling, feature concatenation, and Conv. The integrated feature maps from the neck are passed to detect network for final predictions of fruit target. The detect network houses regression branch that employs both complete intersection-over-union (CIoU) loss function (Zheng et al. [36]) and distribution focal loss (DFloss) (Li et al. [37]) for bounding box loss (Bbloss), and classification (Clsloss) branch that uses binary cross-entropy (BCE) loss. CIoU loss defined by Eq (2) is used to enhance fruit detection performance towards model speed convergence and localization accuracy with special consideration to the overlap area (S), centroid distance (D) and aspect ratio (V) of the predicted box (B) and real box (B^gt^). The DFL depicted in Eq (3) enables probability density and distribution close to the target location, where *s*_*i*_ is sigmoid output, *y*_*i*_ and *y*_*i+1*_ is the interval, *y* is label. For Cls branch, the BCE loss is defined using Eq (4) as *y* is the label for output range (0–1) through sigmoid, and *p(y)* is the predicted probability for all *N* points.


$$\text{O}\in {\text{R}}^{\text{H}\times \text{W}\times (\text{C1}+\text{C2})}=[\text{A}\in {\text{R}}^{\text{H}\times \text{W}\times \text{C1}},\text{B}\in {\text{R}}^{\text{H}\times \text{W}\times \text{C2}}]$$ (1)

$$L_{\text{CIoU}}=\text{S}\left({\text{B},\text{B}}^{\text{gt}}\right)+\text{D}\left({\text{B},\text{B}}^{\text{gt}}\right)+\text{V}\left({\text{B},\text{B}}^{\text{gt}}\right)$$ (2)

$${\text{DFL}}_{\left(s_{i},s_{i+1}\right)}=-((y_{i+1}-y)\text{log}(s_{i})+(y-y_{i})\text{log}(s_{i+1}))\;$$ (3)

$$\text{BCE}=-\frac{1}{N}\sum_{i-0}^{N}y_{i}*\text{log}(p(y_{i}))+(1-y_{i}\text{)}*\text{log}(1-p(y_{i}))\;$$ (4)

Using the input image of 640×640×3 according to Fig 2, the Conv 3×3/2 placed in 0, 1^st^ 26^th^, and 29^th^ layer, and Maxpool found in 4^th^, 10^th^ and 16^th^ layer for downsampling purpose were adopted to effectively reduce large parameters by combining a few convolution kernels and extracts the maximum value of feature map, while ensuring the integrity of the feature information. Similarly, increases the detection speed. The applied Conv 1×1/1 in 2^nd^, 5^th^, 7^th^, 9^th^, 11^th^, 13^th^, 15^th^, 17^th^, 22^nd^, 25^th^, 28^th^, and 31^st^ layer is used for dimensionality reduction, while retaining the salient features from a decrease in the number of feature maps. The case of the Conv 3×3/1 placed in 3^rd^, 6^th^, 12^th^, and 18^th^ layer has similar advantages to Conv 1×1/1 except for its ability to learn features common to different situations for better generalization. The ideal of feature concatenation seen in 8^th^ and 14^th^ of backbone network draws on the experience of Du et al. [38] and Wang et al. [30] to enhance the ability to learn more diverse features by extending the number of channels between 5^th^ and 7^th^ layer, and 11^th^ and 13^th^ layer. This is to increase detection accuracy. The remaining feature concatenation of neck network, placed in 21^st^, 24^th^, 27^th^, and 30^th^ layer enables sharing of information with the backbone network. SPPF at 19^th^ layer enhances the feature maps of the backbone network, while upsampling operations in 20^th^ and 23^rd^ layer is used to increase the resolution of feature maps from low to high in its purest form. Finally, the detect network produces a feature map with dimensions of 80×80, 40×40, and 20×20 through 25^th^, 28^th^, and 31^st^ layer, used to detect fruit targets of different sizes.

In summary, a completely new backbone was designed using the common networks of Conv, Maxpool, feature concatenation and SPPF, and the head network of YOLOv8 having C2f was replaced with Conv to enable fewer parameters, friendly deployment, and faster detection speed of the proposed simplified network for fruit detection.

### Experiment setup

The training and testing of the simplified network, including other YOLO variants, were experimented using YOLOv8.0.40 platform on hardware and environment information stated in Table 2. The network received an input image of 640×640×3 pixels, 9 batch, 0.937 momentum, 0.0005 weight decay, 0.7 IoU, 0.015 hue, 0.7 saturation, 0.4 lightness, 1.0 mosaic, 0.5 scale, 0.1 translate, 7.5 Bbloss, 0.5 Clsloss, 1.5 DFloss, and 100 epochs for training from scratch. In order to deploy the network model on a mobile phone, The developed model format was exported from PyTorch (pt) to open neutral network exchange (onnx) and to ncnn, developed by Tencent. While onnx was designed as an intermediary format between models, ncnn is optimized to run faster than known frameworks on mobile phone CPUs. The ncnn of network model was deployed using ncnn-android-yolov8 [39] on Huawei nova 10 Pro, and compared with other network models. The ncnn was used in this paper for its high-performance neural network inference computing.

**Table 2:** Hardware and environment information.

Hardware	Configure	Environment	Version
System	Ubuntu20.04	Python	3.9.12
CPU	Core i7-12700F	Conda	23.1.0
GPU	RTX3060 (16G)	PyTorch	1.12.1
RAM	32G	CUDA	11.3.1
Hard-disk	1.0T	CUDNN	8.8.0

### Evaluation metrics

Eqs (5)–(10) respectively for precision (P), recall (R), average precision (AP), mean average precision (mAP), speed, and number of parameters (params) are the evaluation metrics used for the networks. TP is the true positive for correct detections, FN is the false negative for missed detections, FP is the false positive for incorrect detections, P(R) means that P is a function of R, AP is the area of curve for a single-class, mAP is the mean of AP values over multi-class, and tends to perform better with an increase, C is the total number of classes, j is the serial number, i is the input-size, k is the convolution kernel-size, o is the output-size and H×W is the size of outputted feature map. Speed is measured in frames per second (fps), params is the network complexity, and layer is the network topology.


$$\text{P}=\frac{\text{TP}}{\text{TP}+\text{FP}}$$ (5)

$$\text{R}=\frac{\text{TP}}{\text{TP}+\text{FN}}$$ (6)

$$\text{AP}=\int_{\text{0}}^{\text{1}}{\text{P}}_{\text{(R)}}\text{dR}$$ (7)

$$\text{mAP}=\frac{\sum_{\text{j}=1}^{\text{C}}{\text{AP}}_{\text{j}}}{\text{C}}$$ (8)

speed=frames/time (9)

$$\text{params}=\left[\text{i}\times \left(\text{k}\times \text{k}\right)\times \text{o}\right]+\text{o}$$ (10)

## Results and discussion

### Performance of networks

**Fig 3:**
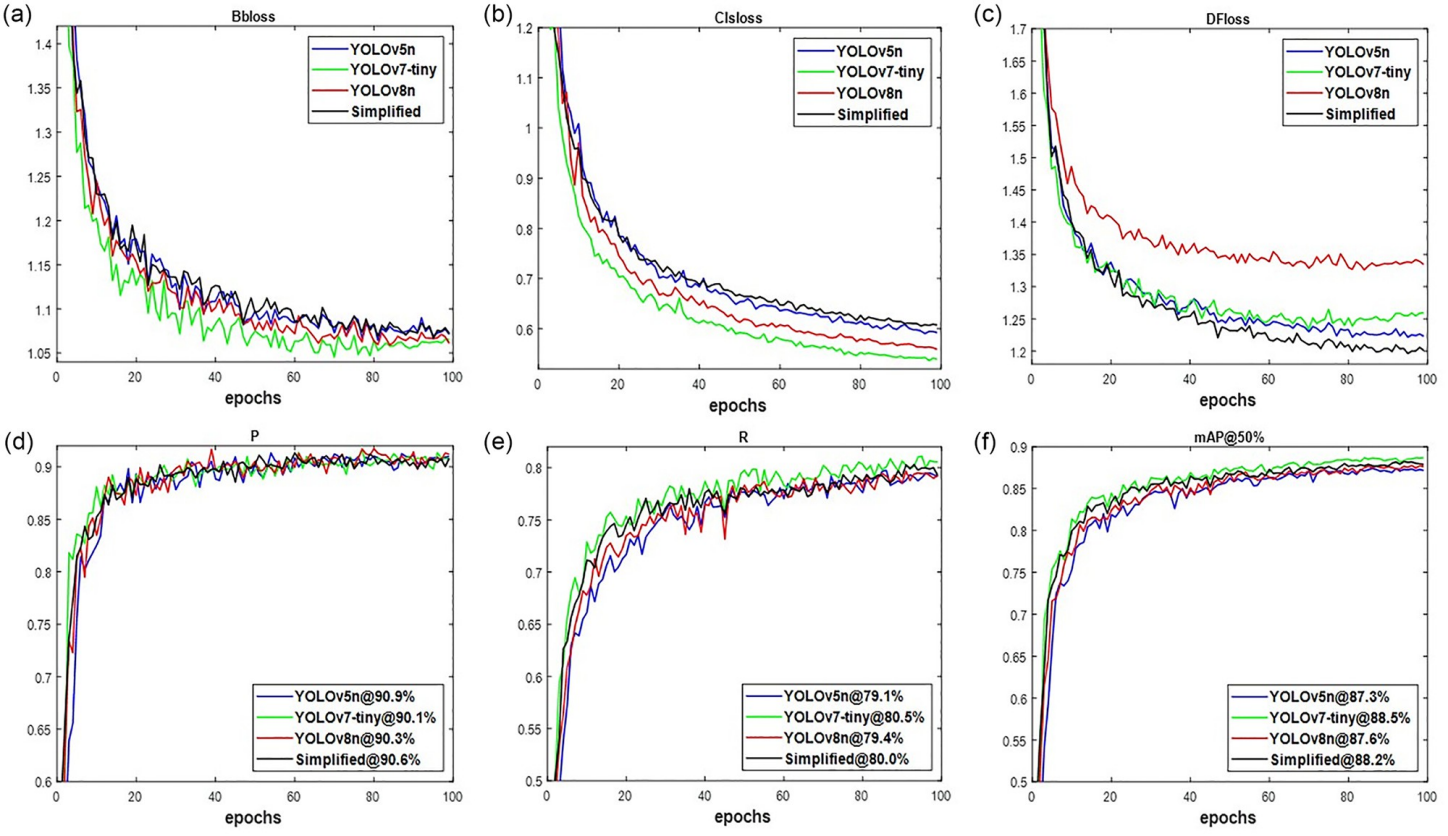
The networks’ output training for (a) Bbloss, (b) Clsloss, (c) DFloss, (d) P, (e) R, and (f) mAP@50%.

The performance of Simplified network in comparison with YOLO-mainstream variants are presented in Fig 3. According to Xu et al. [21], the performance of networks improves with learning process. Using the validation loss attributes, Fig 3(a)–3(c) shows consistence decreasing pattern for all networks. However, the Clsloss of Fig 3(b) is lower than Bbloss of Fig 3(a) and the DFloss of Fig 3(c) to show the level of deeper neural network and error being produced. The level of deeper neural network is measure as YOLOv7-tiny < YOLOv8n < YOLOv5n < Simplified with reference to Bbloss and Clsloss, but not with DFloss. Here, the DFloss of Simplified network is lower than YOLOv5n, YOLOv7-tiny, and YOLOv8n according. Because the Bbloss measures the real location of targets fruit in an image with DFloss combination, resulted to the obtained results displayed in Fig 3(d)–3(f) of P, R and mAP@50%, respectively for valid-set taken from Table 1. The value of Fig 3(d) seen in YOLOv5n is 0.3%, 0.6% and 0.8% higher than Simplified, YOLOv8n and YOLOv7-tiny, respectively, while that of Fig 3(e) noted in YOLOv7-tiny is 0.5%, 1.1% and 1.4% respectively greater than Simplified, YOLOv8n and YOLOv5n. Nevertheless, R is better measure than P in cases where FN are more costly than FP describing how relevant is the number of fruit targets detected. Additionally, having to use mAP@50% of Fig 3(f) is more accurate compare to Fig 3(d) and 3(e). It provides the overall values over multiclass. For this reason, the Simplified network is 0.9% and 0.6% respectively more accurate than YOLOv5n and YOLOv8n, but with a reduced value of 0.3% compared to YOLOv7-tiny. An indication of Simplified network’s superiority over YOLOv5n and YOLOv8n.

The superiority of Simplified network was justified using Table 3. Owning to the percentage difference calculation, the recorded layers of Simplified network at 104 is 59.9%, 54.5%, and 47.1% lower than YOLOv5n at 193, YOLOv7-tiny at 182, and YOLOv8n at 168, respectively. At the same time, the params of Simplified network is 32.6%, 127%, and 50.0% respectively lower than YOLOv5n, YOLOv7-tiny, and YOLOv8n. Meanwhile, the obtained results of tested networks on test-set of Table 1 shows less accuracy compared to valid-set of Fig 3(d)–3(f). The test-set being the unseen data provides an accurate detection performance compared to valid-set. The P of YOLOv5n, YOLOv7-tiny, and YOLOv8n having the same 87.8% is 0.7% larger than Simplified network, whereas in the case of R, the Simplified network is 1.2%, 1.0%, 1.5% more than YOLOv5n, YOLOv7-tiny, and YOLOv8n, respectively. Using the mAP@50%, the Simplified network at 82.4% is 0.4%, -0.2%, and 0.2% respectively more accurate than YOLOv5n, YOLOv7-tiny, and YOLOv8n. YOLOv7-tiny tends to be more accurate than other YOLO-mainstream networks according to Table 3, but with a larger params whose computational cost is very expensive. Therefore, having a less complexity of params and layers with an excellent mAP@50% indicated that the performance of Simplified network is better than YOLOv5n, YOLOv7-tiny and YOLOv8n.

**Table 3:** The networks’ performance with test-set results.

Network	Layers	Params (×10^6^)	Test (P%)	Test (R%)	Test (mAP%)
YOLOv5n	193	2.5	87.8	76.2	82.0
YOLOv7-tiny	182	8.1	87.8	76.4	82.6
YOLOv8n	168	3.0	87.8	75.9	82.2
Simplified	104	1.8	87.1	77.4	82.4

### Count and speed of targets

**Fig 4:**
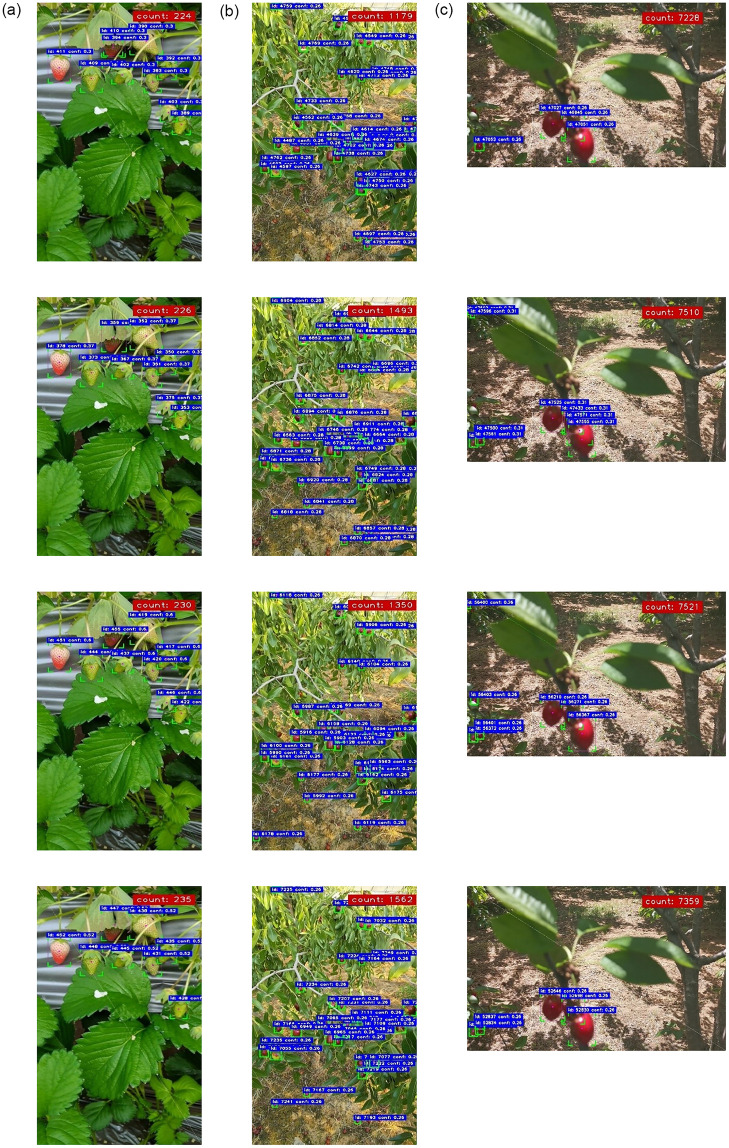
The detected (a) strawberry, (b) jujube, and (c) cherry targets tracked and counted using 1. YOLOv5n, 2. YOLOv7-tiny, 3. YOLOv8n, and 4. Simplified network.

To investigate Simplified network’s level of robustness, it was subjected to track, count and speed of detected targets fruit as displayed in Fig 4 and Table 4, and compared to YOLOv5n, YOLOv7-tiny, and YOLOv8n. Fig 4(a) for tracked strawberry targets and Fig 4(b) for tracked jujube targets show that the number of counts experienced by (4) Simplified network is higher than (1) YOLOv5n, (2) YOLOv7-tiny, and (3) YOLOv8n. In the case of Fig 4(c) for tracked cherry, the number of counts is measure as YOLOv8n > YOLOv7-tiny > Simplified > YOLOv5n. Nonetheless, the overall performance of counts can be analyzed using Table 4. Owning to 1015, 2345, and 5307 frames generated from strawberry, jujube, and cherry, respectively, the average recorded counts for all fruit targets show that the 25.4% of Simplified network is 1.6% and 0.2% higher than 23.8% of YOLOv5n and 25.2% of YOLOv8n, respectively, but with 0.2% lower than 25.6% of YOLOv7-tiny. The obtained higher targets count of YOLOv7-tiny supported the presented results in Table 3 for having the larger params with higher mAP. Furthermore, the detection speed of Simplified network clearly outperformed all the YOLO-mainstream variants, where it is 12.8%, 17.8%, and 11.8% faster than YOLOv5n, YOLOv7-tiny, and YOLOv8n, respectively.

**Table 4:** Summary of track and count with detection speed.

Network	Count				Speed (fps)			
	Strawberry	Jujube	Cherry	(%)	Strawberry	Jujube	Cherry	(%)
YOLOv5n	224	1179	7228	23.8	294	294	303	22.8
YOLOv7-tiny	226	1493	7510	25.6	233	233	227	17.8
YOLOv8n	230	1350	7521	25.2	303	312	312	23.8
Simplified	235	1562	7359	25.4	476	454	455	35.6

### Deployment of networks

**Fig 5:**
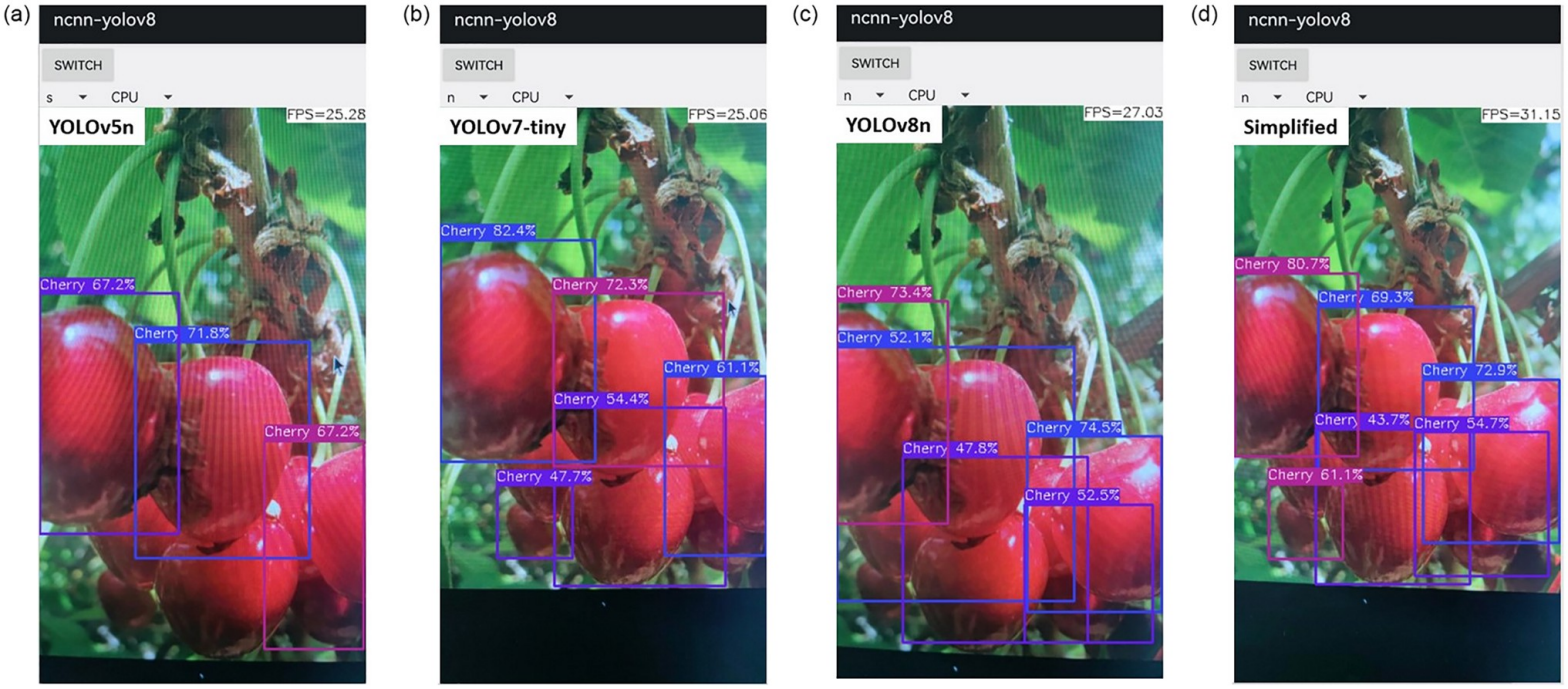
Mobile-phone CPU outputs using network of (a) YOLOv5n, (b) YOLOv7-tiny, (c) YOLOv8n, and (d) Simplified.

The exported networks to the onnx format in Table 5 show that, apart from the similar components found in all networks, the YOLOv5n is associated with further ’add’ and the YOLOv8n is associated with additional ’add’ and ’slice’. Meanwhile, the Simplified network possesses the least numbers of component in terms of Concat, Conv, and Mul, including having the smallest size and export time compared to YOLOv5n, YOLOv7-tiny, and YOLOv8n. The final exported to ncnn from onnx format indicated that under params, Simplified is 58.8%, 63.3%, 53.3% respectively smaller than YOLOv5n, YOLOv7-tiny, and YOLOv8n, while under bin, Simplified is 31.2%, 126%, 48.8% less than YOLOv5n, YOLOv7-tiny, and YOLOv8n, respectively. The obtained results of the deployed ncnn format on mobile-phone are shown in Fig 5. With the exception YOLOv5n, the other networks were able to detect a number of fruit targets on the mobile-phone with an excellent speed. Nevertheless, the detection speed of Simplified network at 31.15 fps is faster than 25.28 fps of YOLOv5n, 25.06 fps of YOLOv7-tiny, and 27.03 fps of YOLOv8n. This is due to its smaller params stated in Table 5, which shared similarity with the presented results in Table 4. With this, the Simplified network is robust, deployable friendly, fast, and able to meet the goal of a computer vision-based understanding of fruit targets in an image.

**Table 5:** Comparison of exported networks.

onnx	YOLOv5n	YOLOv7-tiny	YOLOv8n	Simplified
Add	7	–	6	–
Concat	17	18	17	11
Constant	0	0	0	0
Conv	75	73	63	40
Maxpool	3	6	3	6
Mul	69	67	57	34
Reshape	3	3	3	3
Resize	2	2	2	2
Sigmoid	69	67	57	34
Slice	–	–	8	–
Transpose	1	1	1	1
Size (MB)	9.6	31.0	11.5	7.0
Export (s)	6.3	12.4	4.8	2.0
**ncnn**				
Params (KB)	15.5	16.3	14.6	8.46
Bin (MB)	4.78	15.4	5.74	3.49

### Comparison with other YOLO-variants

Apart from the mentioned YOLO-mainstream variants compared, other existing YOLO variants are noted with complex topology, including for being anchor-based networks. Using Table 6, for instance, Zhang et al. [23] incorporated a ghost network (Han et al. [24]), coordinate attention mechanism (CAM) (Hou et al. [28]) into YOLOv5s to detect a dragon fruit, Qiao et al. [6] added ShuffleNetv2 (Ma et al. [25]) into YOLOv5s to detect and count jujube fruit, Chen et al. [31] improved YOLOv7 with CBAM for citrus detection, and Xu et al. [21] introduced Stem and RCC network into YOLOv5 to detect jujube fruit automatically for ripeness inspection. The Simplified network tends to be easy to understand, with less complexity in comparison. Additionally, its average detection speed taken from Table 4 is faster than that of the proposed network by Fu et al. [12] for kiwifruits, Parico et al. [17] for real-time pear, Lawal [29] for YOLOStrawberry, Fu et al. [40] for YOLO-Banana, Tian et al. [41] and Yan et al. [22] for apples, the YOLOv5-B model by Wu et al. [42] for multi-target recognition of bananas, and so on, as displayed in Table 6. In all, Simplified network is easy to identify, deployable, smaller in parameters, accurate, fast, and generalized against complex environment.

**Table 6:** Performance comparison of YOLO-variant networks.

References	GPU	Weight-size (×10^6^)	Params (×10^6^)	Speed (fps)
Zhang et al. [23]	RTX 3090	11.5	5.2	58.2
Qiao et al. [6]	RTX 3070	1.10	0.4	36.5
Chen et al. [31]	A4000	–	24.3	14.4
Xu et al. [21]	RTX 3060	–	5.2	245.0
Fu et al. [12]	GTX 960M	27.0	–	29.4
Parico et al. [17]	Tesla T4	22.9	–	60.0
Lawal [29]	RTX 3060	3.37	1.7	137.0
Fu et al. [40]	RTX 2070	137.0	–	28.3
Tian et al. [41]	Tesla V100	–	–	3.3
Yan et al. [22]	RTX 2060	12.7	6.5	66.7
Wu et al. [42]	Tesla V100	–	–	111.1
Simplified	RTX3060	3.62	1.8	461.7

## Conclusions and future plans

A Simplified network topology for fruit detection, tracking, and counting was proposed in this paper to enable easy deployment and understanding, accuracy, speed, fewer parameters, and generalization against complex natural environment. It’s imbibed common networks of Conv, Maxpool, feature concatenation and SPPF, and build upon YOLOv8 framework. More so, it was validated on a robust fruit image dataset of dense targets. The aggregated detection results of Simplified network in terms of params, layers, speed, mAP@50%, counting, and deployment outperformed the mainstream networks of YOLOv5n, YOLOv7-tiny, and YOLOv8n. However, it shows an insignificant reduction in mAP@50% and counting compared to YOLOv7-tiny. This is due to the larger params recorded for YOLOv7-tiny. Future investigations will require improvements in detection accuracy, the modification of SPPF with a simpler network in the topology, and the detection performance of another converted format on mobile-phones.

## Supporting information

S1 Data (ZIP)
