# Supplementary figures and images for: A simplified network topology for fruit detection, counting and mobile-phone deployment
Source: PLoS One. 2023 Oct 9;18(10):e0292600. doi: 10.1371/journal.pone.0292600 (PMC10561836; doi:10.1371/journal.pone.0292600)

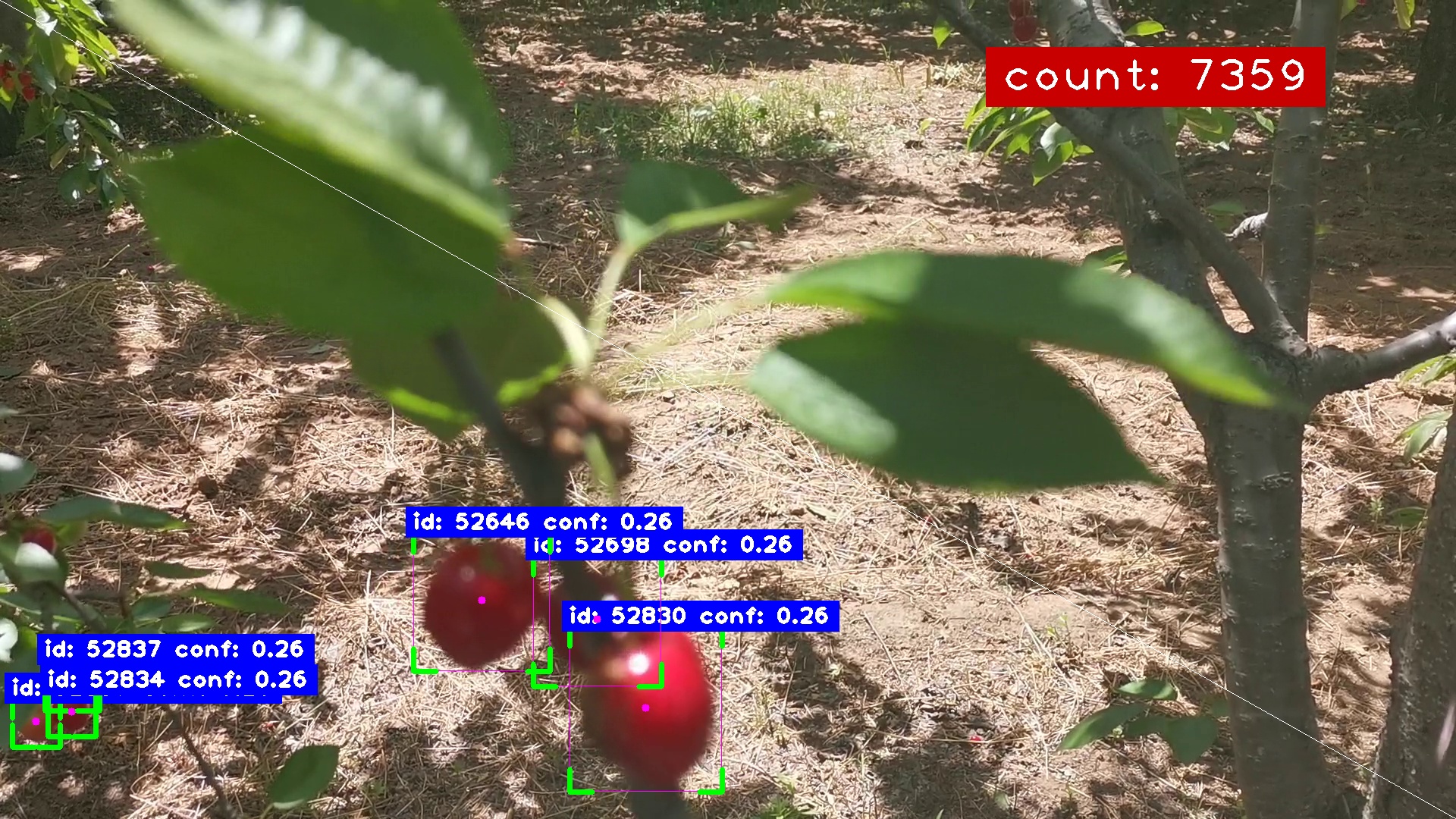

Supplement: S1 Data — (ZIP) [file pone.0292600.s001.zip › Simplified/c.jpg]

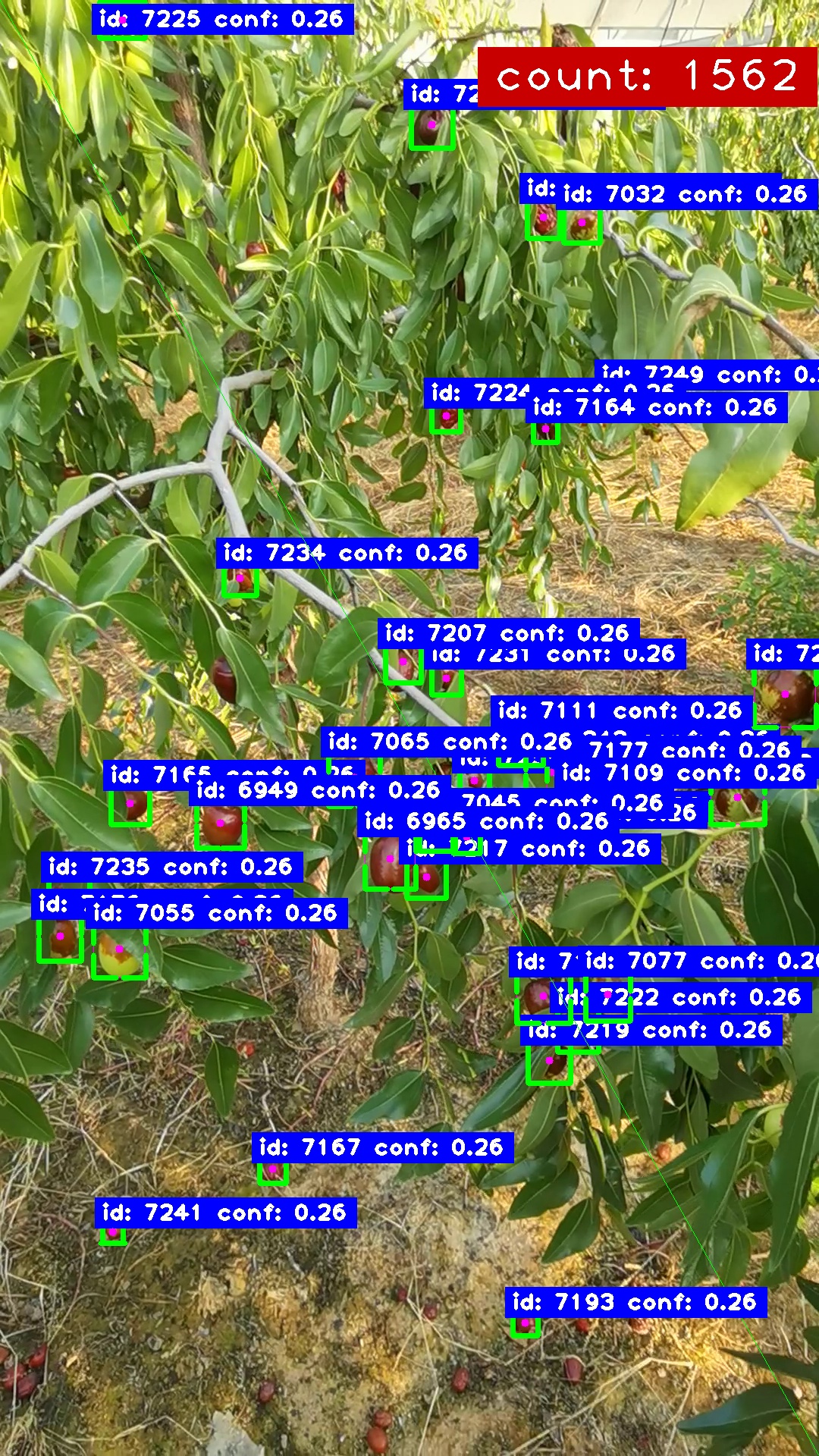

Supplement: S1 Data — (ZIP) [file pone.0292600.s001.zip › Simplified/j.jpg]

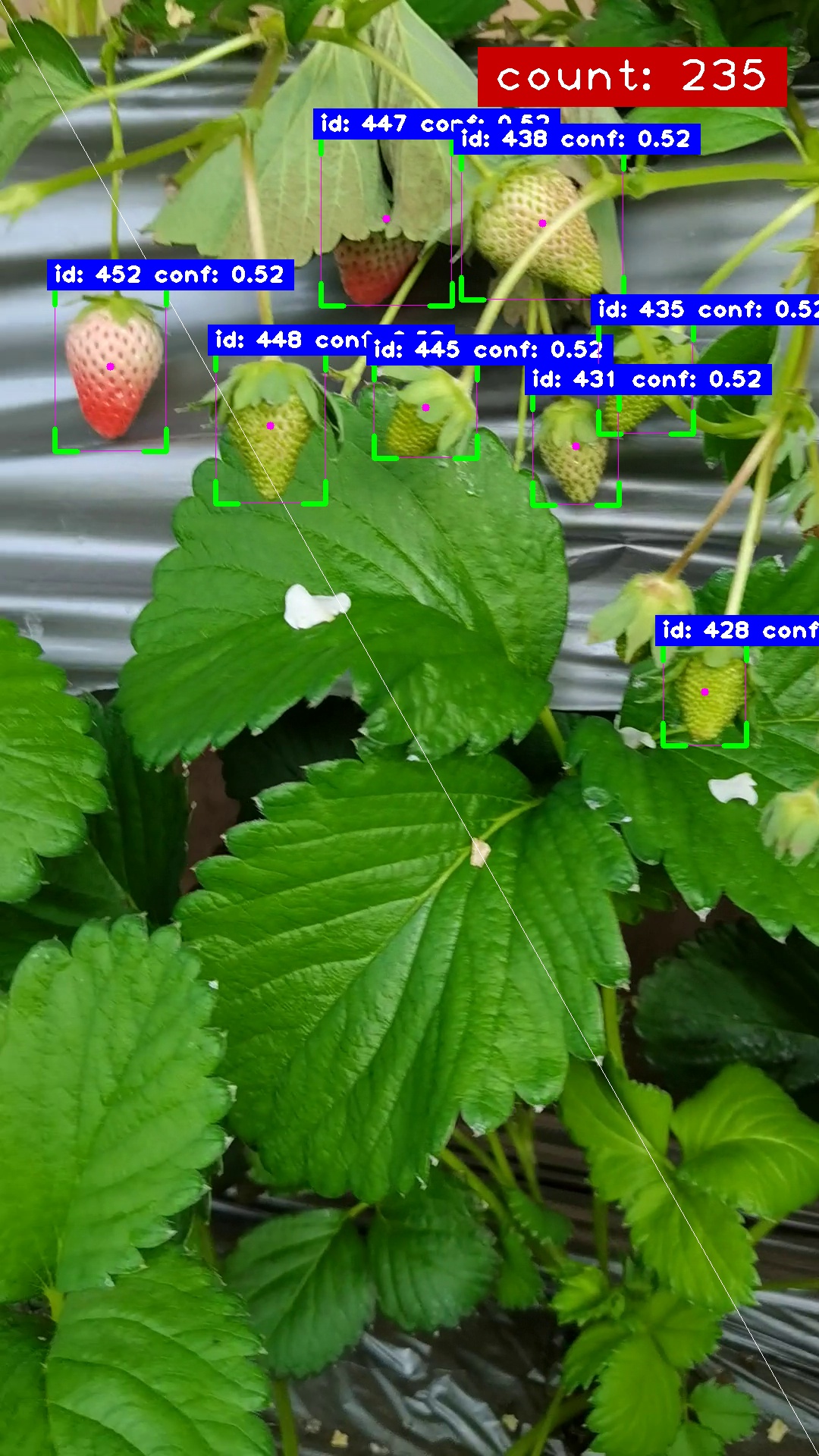

Supplement: S1 Data — (ZIP) [file pone.0292600.s001.zip › Simplified/s.jpg]

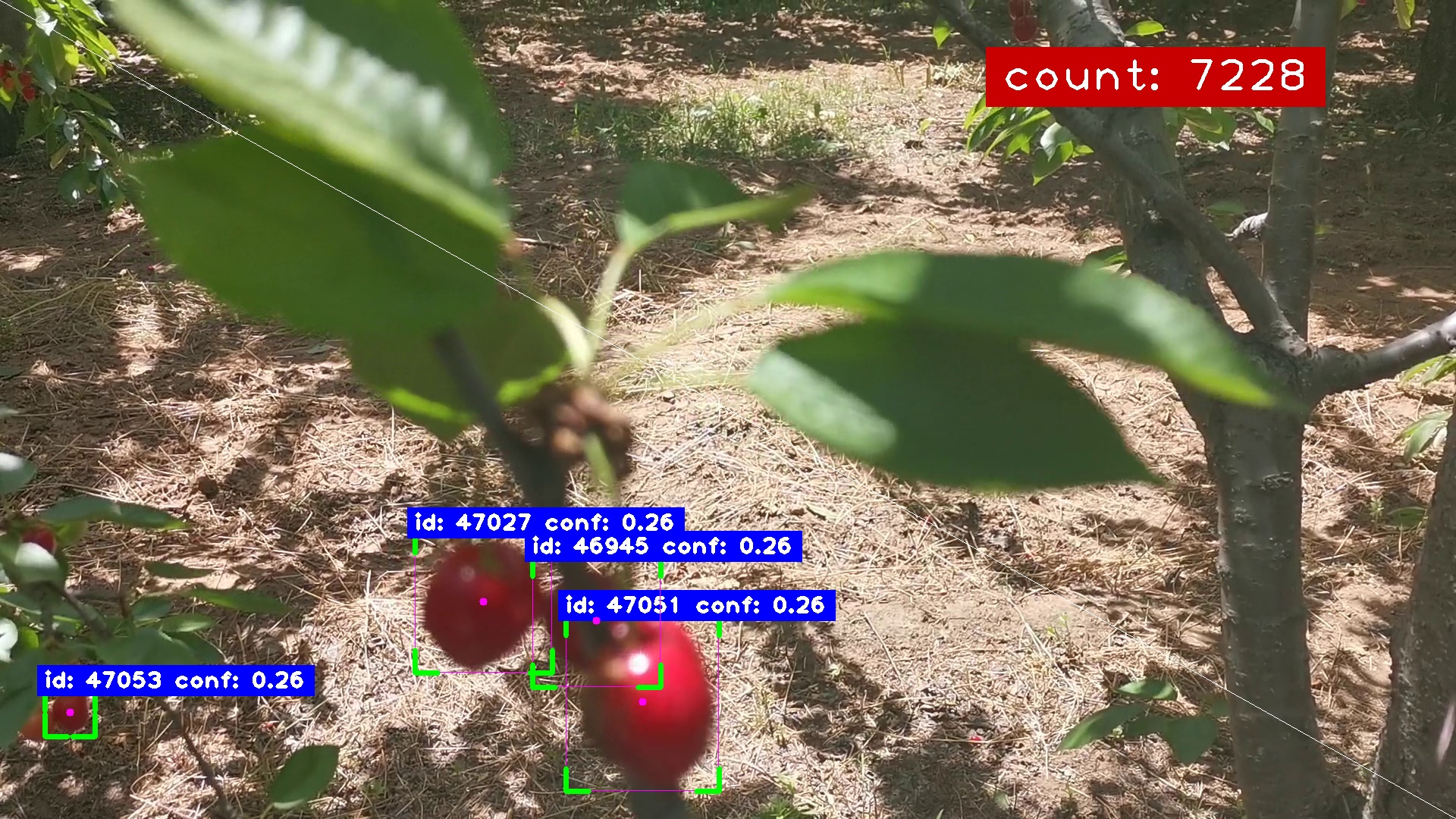

Supplement: S1 Data — (ZIP) [file pone.0292600.s001.zip › yolov5n/c.jpg]

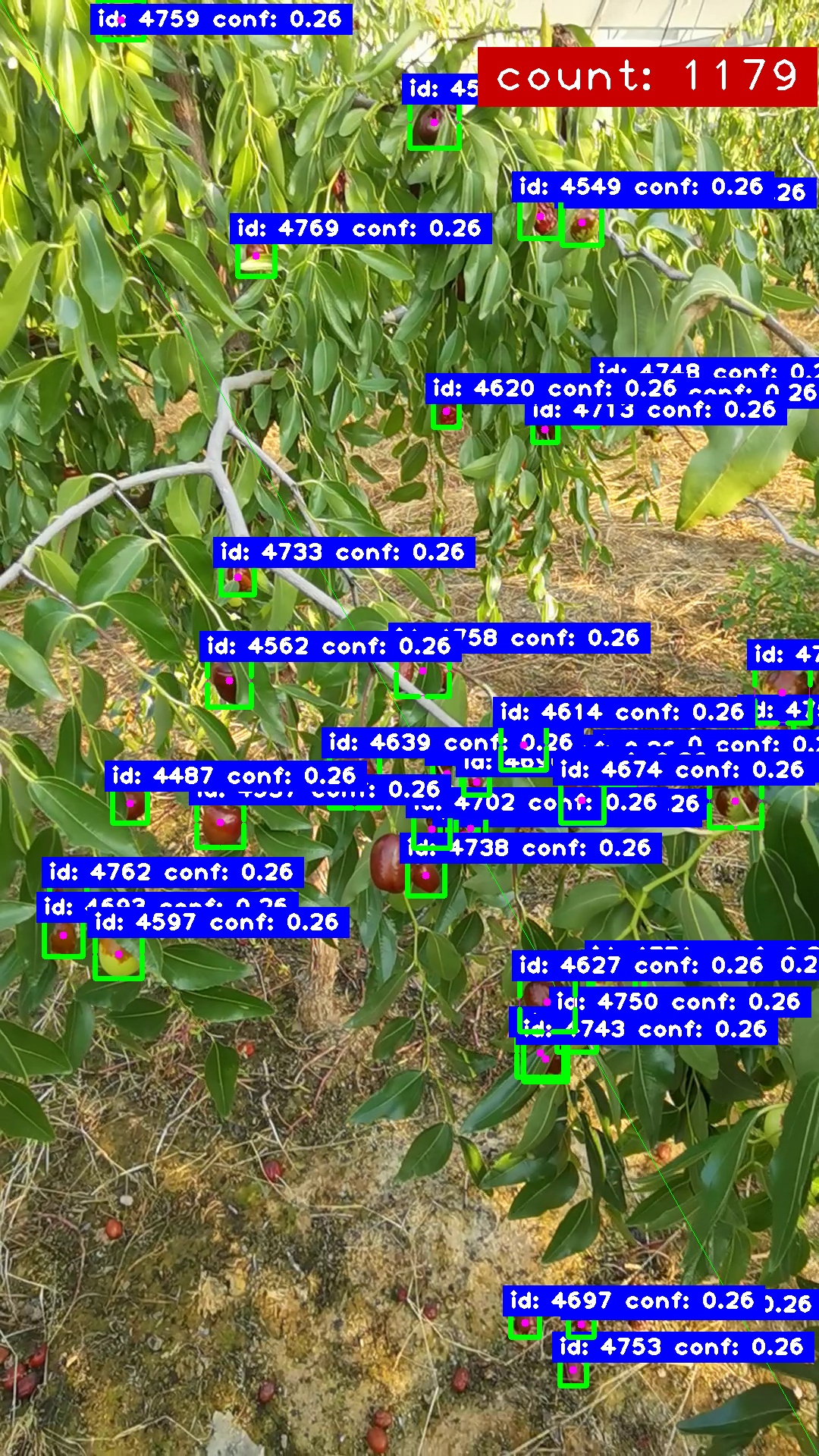

Supplement: S1 Data — (ZIP) [file pone.0292600.s001.zip › yolov5n/j.jpg]

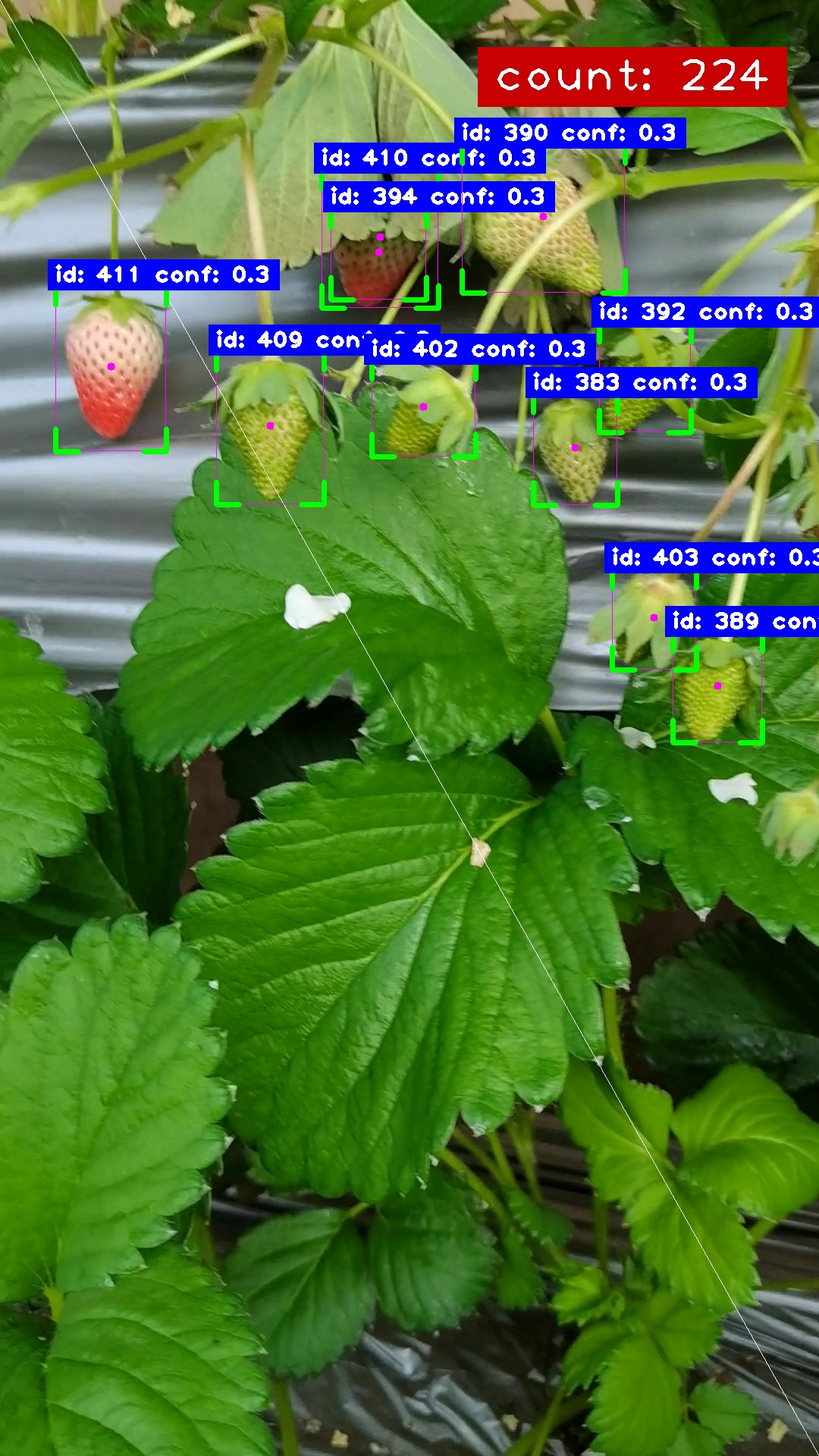

Supplement: S1 Data — (ZIP) [file pone.0292600.s001.zip › yolov5n/s.jpg]

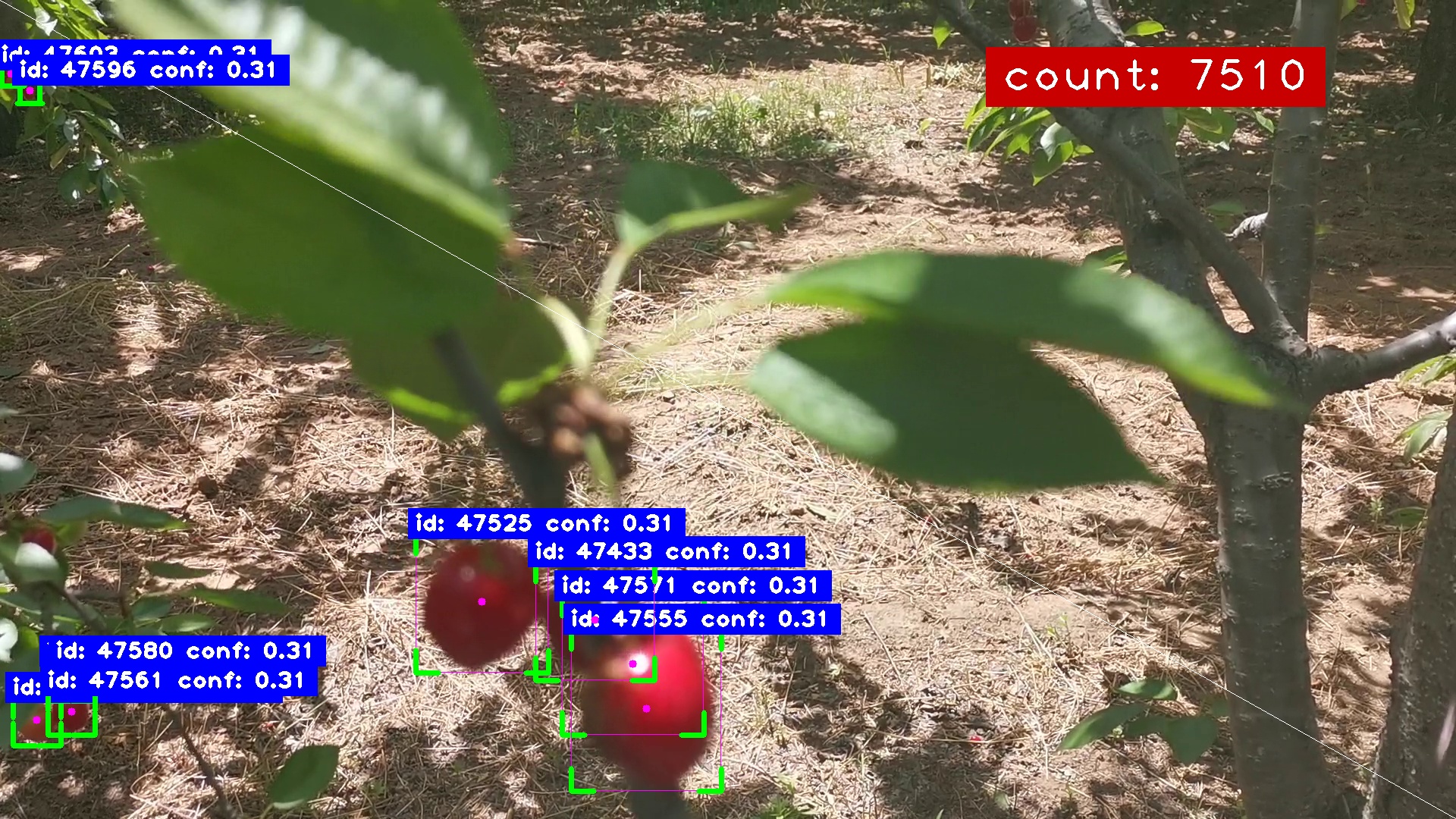

Supplement: S1 Data — (ZIP) [file pone.0292600.s001.zip › yolov7-tiny/c.jpg]

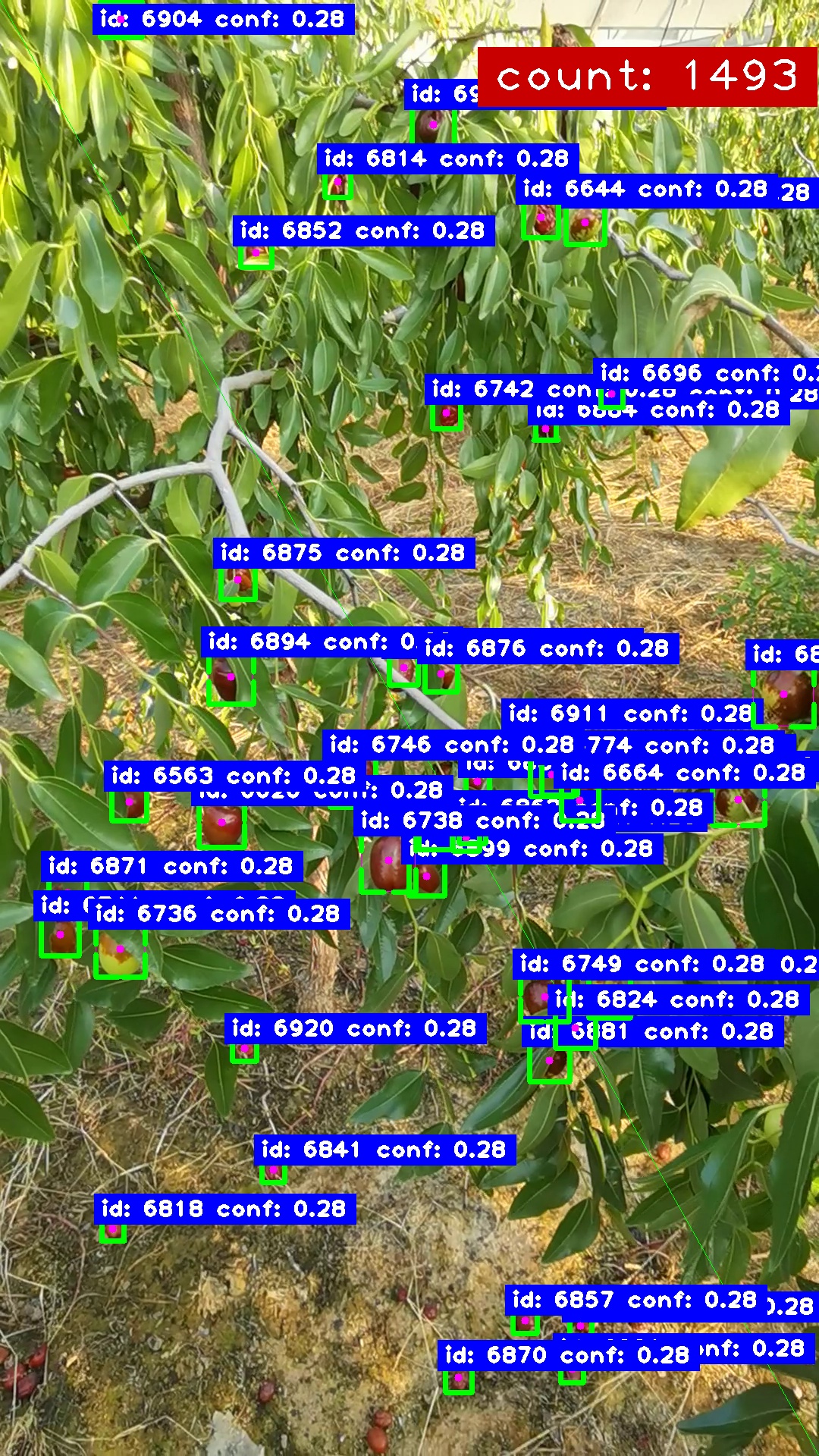

Supplement: S1 Data — (ZIP) [file pone.0292600.s001.zip › yolov7-tiny/j.jpg]

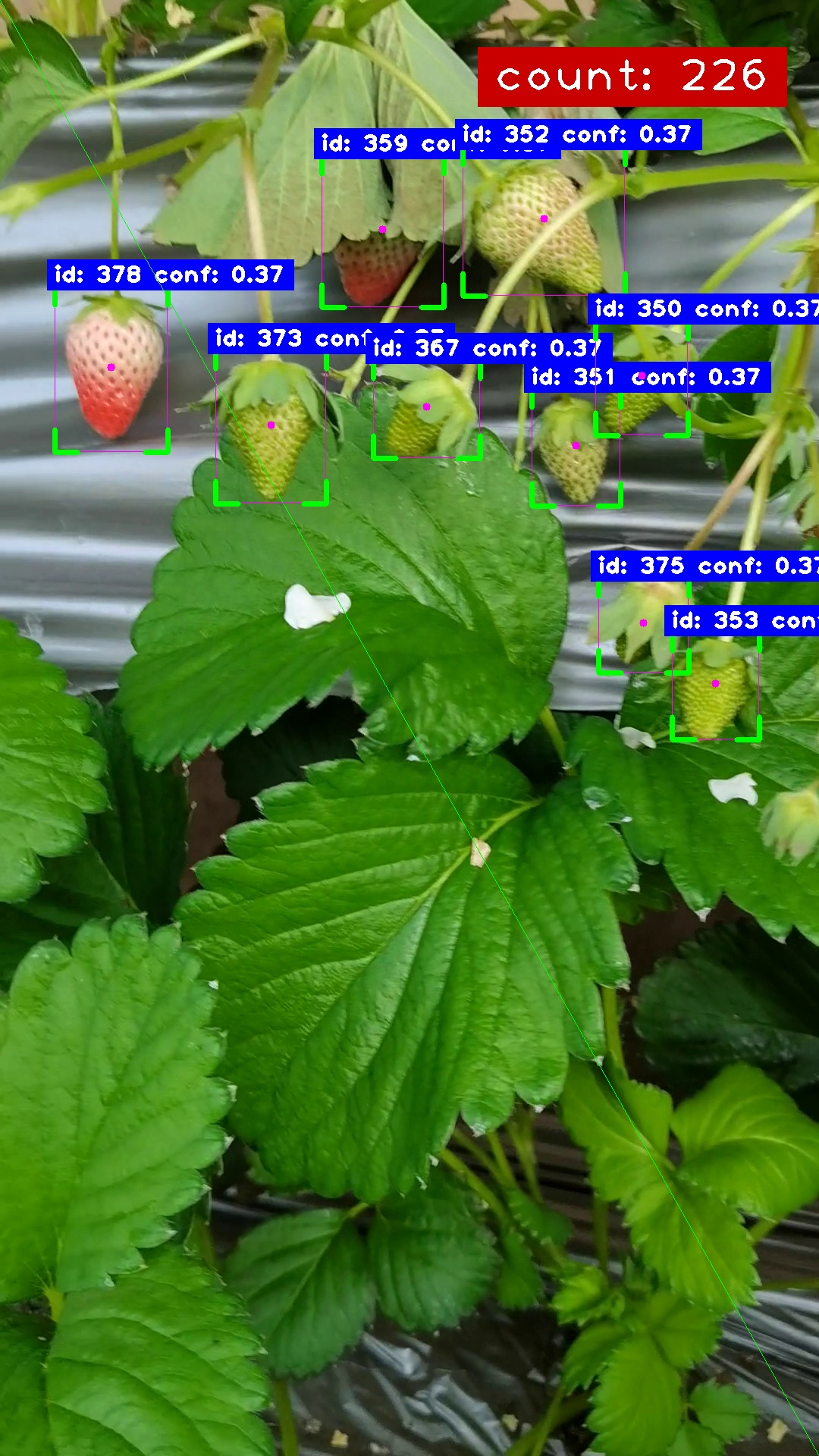

Supplement: S1 Data — (ZIP) [file pone.0292600.s001.zip › yolov7-tiny/s.jpg]

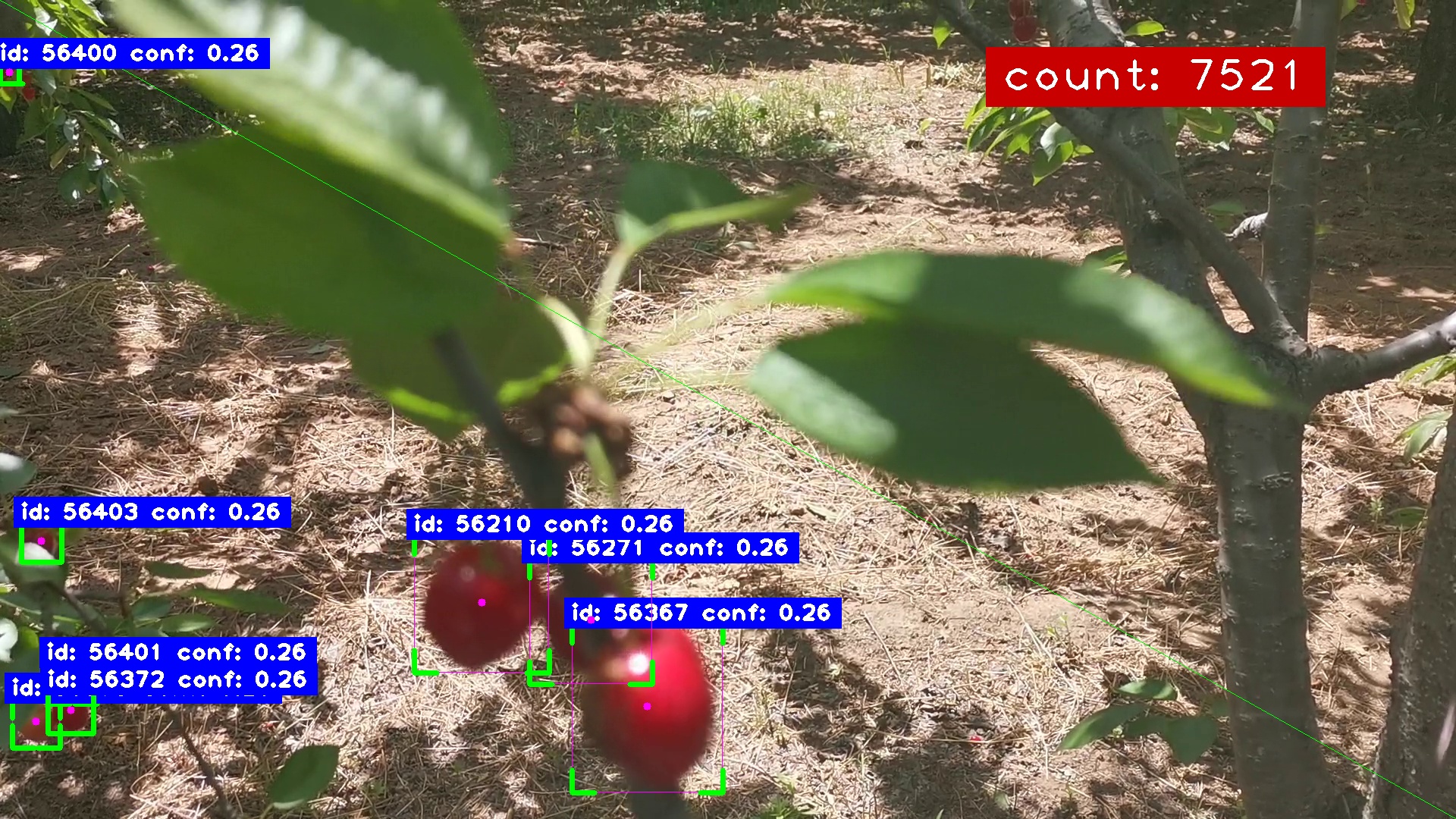

Supplement: S1 Data — (ZIP) [file pone.0292600.s001.zip › yolov8n/c.jpg]

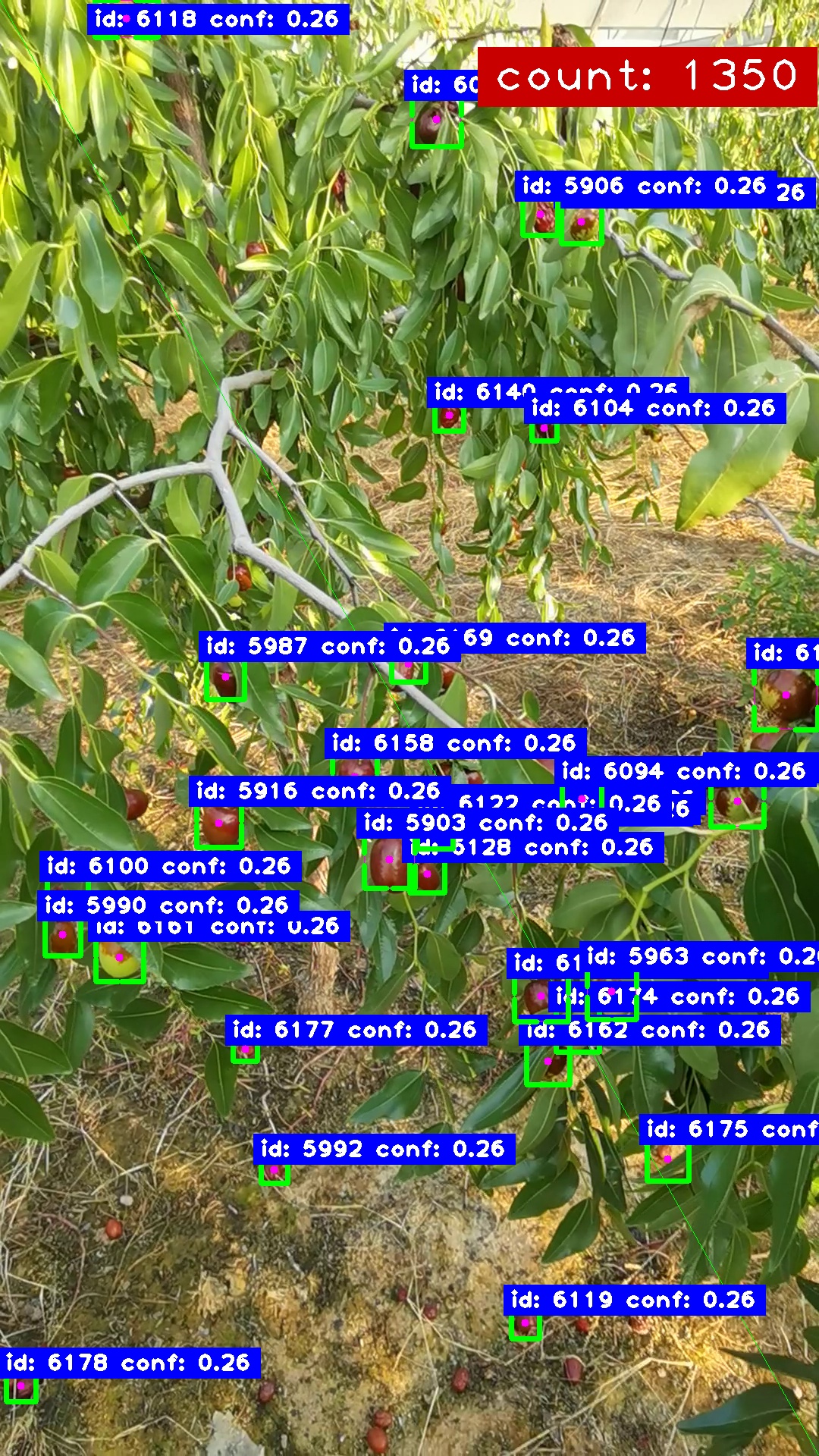

Supplement: S1 Data — (ZIP) [file pone.0292600.s001.zip › yolov8n/j.jpg]

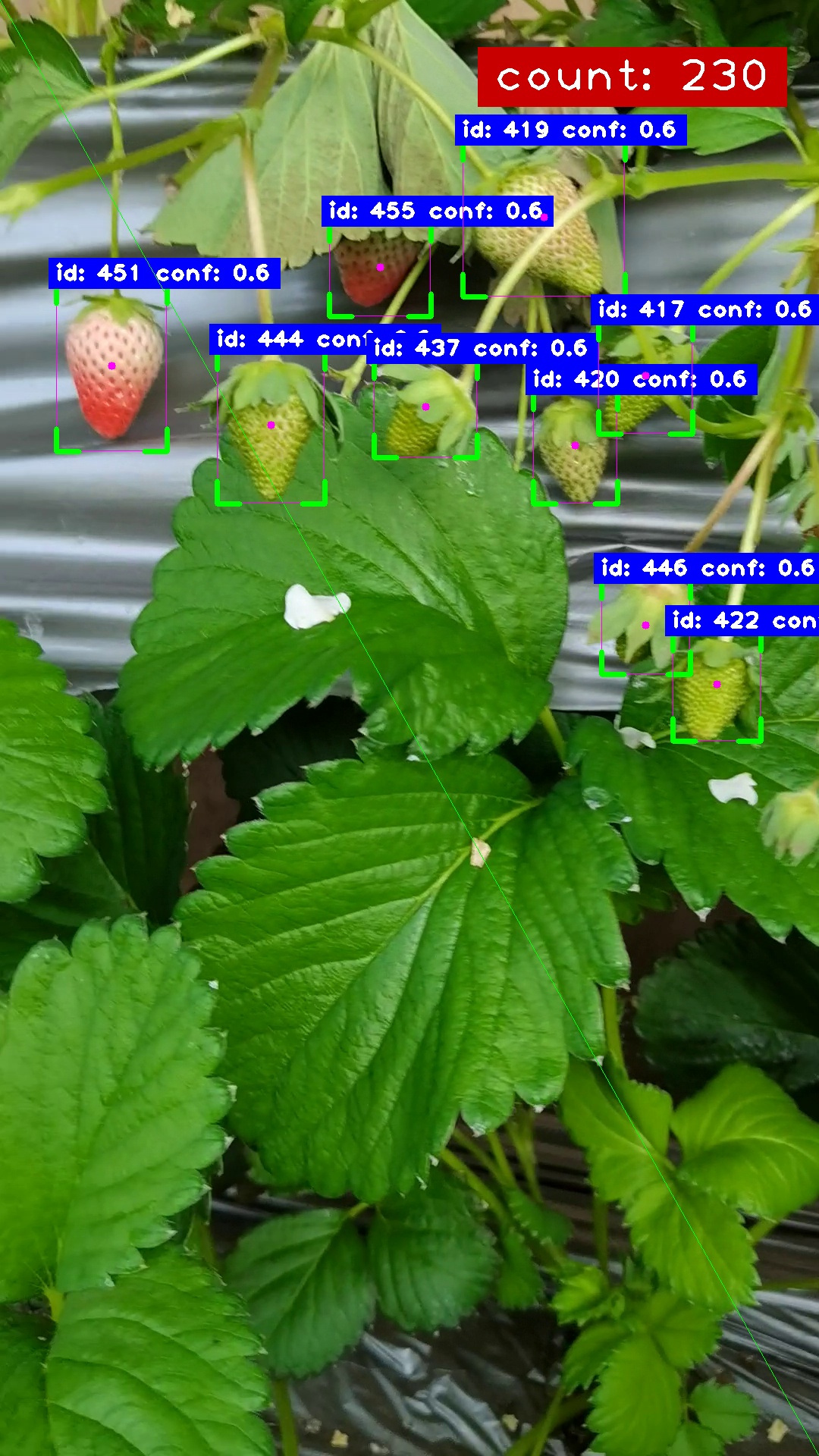

Supplement: S1 Data — (ZIP) [file pone.0292600.s001.zip › yolov8n/s.jpg]
